# Effects of tactile vibration feedback system on balance function and walking ability of a unilateral transtibial amputee with a prosthesis: A case report

**DOI:** 10.1097/MD.0000000000022450

**Published:** 2020-09-25

**Authors:** Shi-Qi Wang, Ya-Qian Gao, Ze-Hua Xu, Fang-Yuan Xu, Li Yuan

**Affiliations:** Rehabilitation Medicine Department, The Affiliated Hospital of Southwest Medical University, Luzhou, Sichuan, People's Republic of China.

**Keywords:** amputee, case report, feedback, vibration

## Abstract

**Rationale:**

There is still a lack of case reports about tactile vibration feedback devices for the treatment of transtibial amputees so far. This case report aims to introduce a tactile vibration feedback device designed to improve the balance and walking function of the transtibial amputee.

**Patient concerns:**

The amputee was a 20-year-old man with right transtibial amputation in a car accident four years ago.

**Diagnose:**

The clinical diagnosis of him was “Right transtibial amputation,” and the rehabilitation diagnosis was “Motor dysfunction (Balance function abnormality and Gait abnormality).”

**Interventions:**

The patient was reminded to adjust their posture in time via the tactile vibration feedback device.

**Outcomes:**

The balance and walking function of the volunteer transtibial amputee was improved.

**Conclusion:**

The tactile vibration feedback device has the potential to improve the balance and walking function of the transtibial amputee after installation. Potential fields that can be recommended for future research include intelligent prosthetics, feedback training, motor function, prosthetic acceptance, compliance, social communication, and the quality of life.

## 1 Introduction

Sensory feedback therapy is a research hotspot in the field of the prosthesis. Tactile feedback from the lower extremities is essential for maintaining balance, completing walking, and performing complex daily activities.^[1]^ In the case of transtibial amputation, amputees will lose part of his or her sensory and motor function.^[2]^ Besides, most prosthetics are unable to correctly and comprehensively perceive and feedback the motor information of lower limbs, and can not effectively make up for the loss of sensation caused by the loss of the limb.

Previous literature has reported that using tactile vibration feedback to treat upper-limb amputees and achieved active results.^[3]^ Husman M. A. has invented a wearable skin stretch haptic feedback device, which has been shown to have the potential to improve balance control in amputees.^[4]^ However, his device was only tested on normal people. We do not know the effect of the device on amputees.

At present, there is still a lack of case reports about tactile vibration feedback devices for the treatment of transtibial amputees so far. This case report introduced a tactile vibration feedback device designed by our team, aiming to improve the balance and walking ability of the transtibial amputee. Of note, written informed consent was obtained from the patient to publish the case report and accompanying images.

## 2 Case report

### 2.1 Patient information

The tactile vibration feedback device was tested on a volunteer transtibial amputee. The amputee was a 20-year-old man with right transtibial amputation in a car accident 4 years ago. He weighed 62.5 kilograms (kg). The length from the medial tibial plateau to the stump was 18.2 centimeters (cm), and the prosthesis has been worn for 4 years. The amputee was in good health and had no history of hypertension or diabetes. The amputee did not receive formal rehabilitation training before participating in the test. When the amputee walked, his gait was significantly different from that of normal people. The clinical diagnosis of him was “Right transtibial amputation” and the rehabilitation diagnosis was “Motor dysfunction (Balance function abnormality and Gait abnormality)”. He signed the informed consent form and participated in the test after we introduce the purpose and method of the intervention for him. He was not allowed to use alcohol or painkillers a week before the test to ensure that the stump sensory was normal. The Clinical Trial Ethics Review Committee of the Affiliated Hospital of Southwest Medical University approved this study (number: KY2019160).

### 2.2 Therapeutic intervention

**Figure 1:**
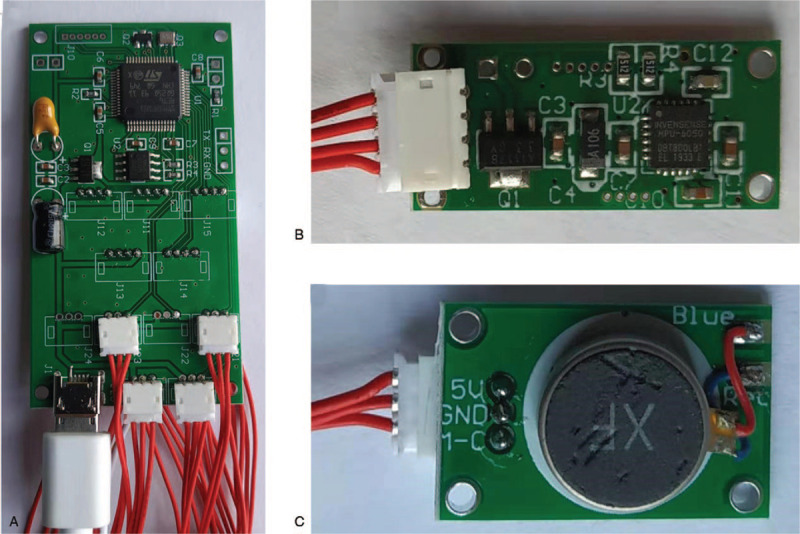
Composition of the tactile vibration feedback device. (A) Control motherboard. (B) Gyroscope sensor. (C) Vibration motor.

**Figure 2:**
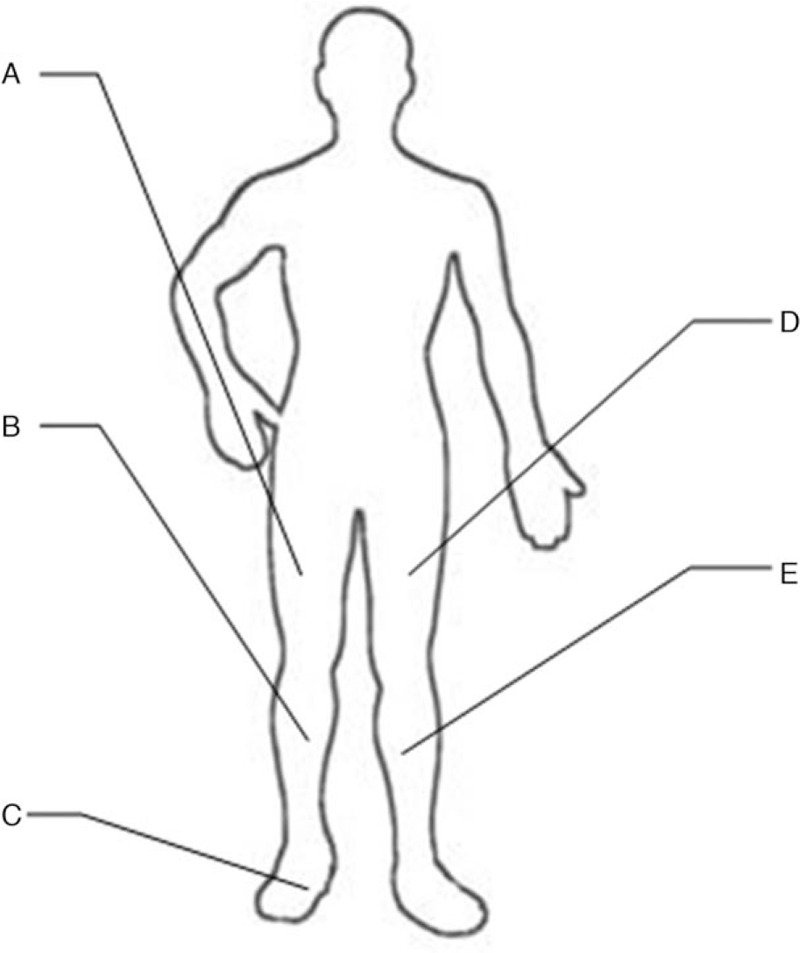
Schematic diagram of the device installation position. Prosthetic side: (a) A gyroscope sensor and 4 vibration motors around the middle part of the thigh. (b) A gyroscope sensor at the lower leg. (c) An artificial foot plane sensor. Healthy side: (d) A gyroscope sensor at the thigh. (e) A gyroscope sensor at the lower leg.

The tactile vibration feedback device consisted of a control motherboard, 5 gyroscope sensors, and 4 vibration motors (Fig. 1). The sensors were located on both thighs and lower legs, as well as on the dorsum of the artificial foot. The vibration motors were located around the middle part of the thigh (front, back, inside, and outside). The whole device was powered by a portable mobile 10000mAh battery and was tied to the waist together with the control motherboard. Figure 2 shows the location where each component is installed.

Five gyroscope sensors and four vibration motors were connected to the control motherboard via a silicone wire. The data (rotation angle) collected by the gyroscope sensor was transmitted to the CPU through five serial ports for processing. When the difference between the angles was greater than 5, the device would compare the motion of the prosthetic limb with that of the healthy limb, and then the corresponding part would be stimulated by the vibration motor. When the motion of the prosthetic side was large, the control motherboard guided the vibration motor to stimulate the front side of the corresponding limb. Oppositely, when the action motion was small, the vibration motor stimulated the posterior side. In this way, the amputee was reminded to adjust their posture in time.

### 2.3 Evaluation and outcome

Before using the device, the amputee was evaluated by Tinetti Performance Oriented Mobility Assessment (Tinetti POMA), indoor gait analysis, and outdoor 1000 m complex pavement test.^[5,6]^

The Tinetti POMA could be used to evaluate the gait and balance function of amputees. The total score is 28. The lower the score, the worse the balance. The amputee walked 3 times at a comfortable pace in the GAITRite gait analysis system (GAITRite Gold, CIR Systems, PA, USA) to record the gait-related parameters. The outdoor 1000 m complex pavement test used our hospital road as the test site, including flat road, grass, ramp, and staircase. The pedometer and stopwatch were used to record the steps, energy consumption, and the total time.

**Figure 3:**
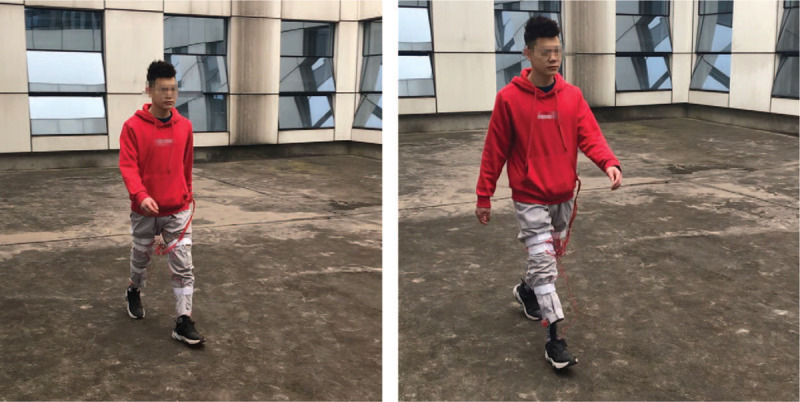
The subject walked after installation.

The amputee installed the device with a therapist help and was evaluated again after a three-hour familiarization process (Fig. 3). No adverse and unanticipated events occurred. The patient was very satisfied with the process of treatment and evaluation. Table 1 shows that the Tinetti POMA score increased before installation, especially the gait score. The parameters of indoor gait analysis were improved, and the number of steps, total time, and energy consumption in outdoor 1000 m complex road test decreased. The results suggested that the balance and walking function of the volunteer transtibial amputee could be improved.

**Table 1:**
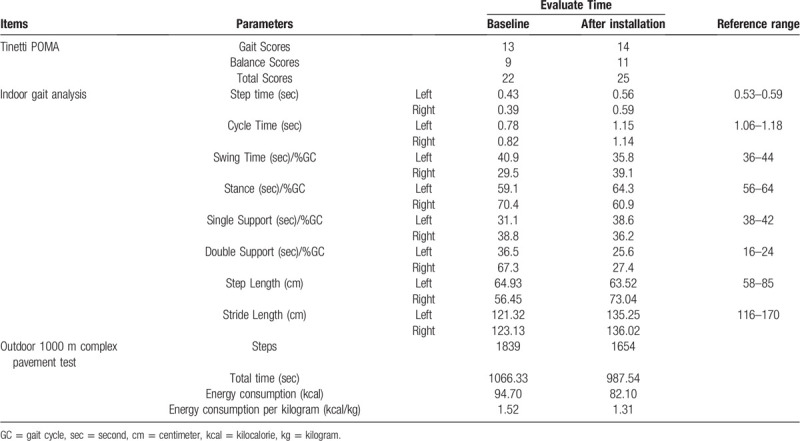
Evaluation results.

## 3 Discussion

After amputation, it is necessary to wear prosthetics and carry out the functional exercise as soon as possible to achieve the purpose of early rehabilitation. However, after wearing traditional prosthetics, there are still differences between the balance and walking function of amputees and normal people. The wrong balance and gait characteristics would affect the comfort of the stump, reducing the walking efficiency. The wrong posture may cause secondary physical damage to the amputee over time.^[7]^ Therefore, the improvement of balance and walking function is vital to transtibial amputees with prostheses.

Duclos C vibrated the lateral neck or hip muscles of lower limb amputees and found that the muscle vibration in both positions solved the problem of posture asymmetry in more than half of amputees.^[8]^ This indicated that muscle vibration could maintain or cause directional changes in posture. Raveh E found that when visual feedback was disturbed, the increase of tactile vibration feedback in prosthesis users had a positive impact on the performance and accuracy of the prosthesis.^[9]^ Although the subjects in this study were patients with upper limb amputation, it was undeniable that tactile vibration feedback has a positive effect on the improvement of amputee's movement and function. And our case report showed the tactile vibration feedback device has the potential to improve the balance and walking function of transtibial amputees after installation, which also shows a good development prospect.

Fan RE designed a pneumatic haptic feedback system, being able to provide sensory feedback to the lower extremities, but his device did not compare the data collected by the sensor with the healthy side in real-time.^[10]^ As for our device, CPU compared the data between the healthy limb and the prosthetic limb, and the data between the foot plane and the preset data, after collecting the data transmitted by the sensor. According to the results of the data comparison, the amputee was guided to change their posture by vibrating motor.

Our device had highly sensitive sensors. The sensors mainly used gyroscope acceleration sensor chip (type: MPU6050). The sensor weighed 1 gram (g), was 2.5 cm long and 1 cm wide. It was an integrated motion 6-axis motion processing component that could record the motion angles of the lower limbs in all directions and could reduce installation space. The control motherboard weighed 10 g, was 6.5 cm long and 3.4 cm wide. The CPU model used by the sensor and control motherboard is STM32F103RET6, with fast data processing capability. The vibration motor weighed 1.5 g, was 1.9 cm long and 1.3 cm wide. The total weight of the whole device is only 21 g.

In addition, the foot plane sensor was used to determine whether the amputee was making a turn. Even normal lower limbs could not fully coordinate the movements of the 2 limbs when making a turn. In order to avoid erroneous instructions, the vibrating motor suspended the stimulation to the patient when he was detected to make a turn. When the patients foot plane was inverted or everted, the sensor could detect the abnormal position of the artificial foot, and the vibration motor would stimulate the inside and outside of the thigh to remind the patient of the posture problem of the foot plane.

This simple technique can be mounted on the already used prosthesis with no change of prosthetic components. It is also expected to be directly integrated with prosthesis design in the future. Potential fields that can be recommended for future research include intelligent prosthetics, feedback training, motor function, prosthetic acceptance, compliance, social communication, and the quality of life.

There were some limitations in this case report. First, the battery life of the device was unstable. The power consumption of the whole device was about 25 mA and could last for more than 360 hours, but the more exercise of the amputee, the more power consumed. Users had to charge the device within half a month. Further consideration should be given to expanding the capacity of the portable mobile battery or changing to button batteries for easy replacement. Second, this device only tested on one amputee, and further high-quality clinical research was needed. In addition, the intervention time and follow-up time for the patient in this study were short, which could be appropriately extended in the future to observe the long-term effect of the device.

## 4 Conclusion

In summary, the tactile vibration feedback device has the potential to improve the balance and walking function of the transtibial amputee after installation. Potential fields that can be recommended for future research include intelligent prosthetics, feedback training, motor function, prosthetic acceptance, compliance, social communication, and the quality of life.

## Author contributions

**Device and study design:** Fang-Yuan Xu, Li Yuan.

**Investigation:** Shi-Qi Wang, Ya-Qian Gao.

**Methodology:** Shi-Qi Wang, Ya-Qian Gao, Ze-Hua Xu.

**Project administration:** Fang-Yuan Xu, Li Yuan.

**Resources:** Ze-Hua Xu.

**Writing – original draft:** Shi-Qi Wang.

**Writing – review & editing:** All authors.
